# Exopolysaccharide, Isolated From a Novel Strain *Bifidobacterium breve* lw01 Possess an Anticancer Effect on Head and Neck Cancer – Genetic and Biochemical Evidences

**DOI:** 10.3389/fmicb.2019.01044

**Published:** 2019-05-09

**Authors:** Lin Wang, Yifei Wang, Qingxiang Li, Kaiyue Tian, Le Xu, Guorong Liu, Chuanbin Guo

**Affiliations:** ^1^Department of Oral and Maxillofacial Surgery, Peking University School and Hospital of Stomatology, Beijing, China; ^2^Department of Oral and Maxillofacial Plastic and Trauma Surgery, Beijing Stomatological Hospital, School of Stomatology, Capital Medical University, Beijing, China; ^3^Beijing Engineering and Technology Research Center of Food Additives, Beijing Technology and Business University, Beijing, China

**Keywords:** *Bifidobacterium*, exopolysaccharide, genome, biosynthesis, anticancer, probiotic, functional food

## Abstract

Probiotic bacteria exopolysaccharides (EPS) have been recognized as molecules that regulate immune development and have anti-inflammation and anticancer effects. Yet, these bioactivities are of interspecies diversity; thus, examining the gene clusters of EPS and biosynthesis pathways are essential for selecting the better application of specific EPS. In this study, we isolated a new *Bifidobacterium* strain, named *B. breve* lw01. A complete genome of *B. breve* lw01 was sequenced revealing a circular 2,313,172 bp chromosome. Furthermore, a deep excavation of genome sequence from different database based on the comparison-selected results was performed to explore the gene cluster responsible for EPS synthesis. We found that *B. breve* lw01 harbors a new EPS-encoding cluster with 14 predicted genes, which could be divided into three groups according to the biosynthesis pathway hypothesis. Using tertiary purification, high purity EPS were obtained. EPS is composed of rhamnose (Rha), arabinose (Ara), galactose (Gal), glucose (Glc), and mannose (Man) in a molar ratio of 0.35:0.44:1.38:0.67:1.65. With reference to its bioactivity, it showed to possess anticancer activity against Head and Neck Squamous Cell Carcinoma cell line by regulating cell cycle arrest and cell apoptosis promotion. To sum up, this study examined the biosynthesis and bioactivity of EPS using a new isolated *B. breve* strain, which could be used to clarify its further application in functional food or drug industry.

## Introduction

Microbial EPS are carbohydrate polymers surrounding the envelope of most bacteria. The bacterial EPS has initially gained lots of attention from researchers for its effect on biofilm or capsular polysaccharides formation that act as virulence factors in infectious diseases (Gonzalez-Escobedo et al., 2011; Yamanaka et al., 2011). Recently, bacterial EPS have re-gained attention for their implication on human health, especially since bacteria with some probiotic traits and their EPS could contribute to the host health maintenance. Among these, *Lactobacillus* and *Bifidobacterium* are mostly used in the formulation of probiotic foods or supplements for human consumption.

*Bifidobacterium*, which is one of the dominant early colonization bacteria in human intestinal tract, was proven to regulate immune development (Arboleya et al., 2012), immune modulation (Fanning et al., 2012), and to possess an anti-inflammation (Strisciuglio et al., 2015) and anticancer (Wang et al., 2017) activity. The immune response is stain-specific (Lopez et al., 2010) and one of the mechanisms driving the immune functions is regulated by bifido-EPS. Bifido-EPS may facilitate *Bifidobacterial*-host interaction through immune modulation (Fanning et al., 2012) and provide pathogen protection through a physical barrier, also known as “*Bifidobacterial* biofilm” (Ruas-Madiedo et al., 2010). In addition, bifido-EPS has shown to possess an antioxidant activity, which may further reduce tissue damage (Li et al., 2014). In addition, EPS can inhibit DNA synthesis (You et al., 2004) and the expression of gene involved in angiogenesis. Yet, these bioactivities, which rely on the related physicochemical characteristic, are of interspecies diversity. Thus, for better application of specific bifido-EPS, gene clusters, chemical composition and its hypothetical biosynthesis pathways prediction need to be carefully examined.

With regards to chemical composition and its synthesis method, HePS, which are type of the EPS polymers reported to be the unique form in *Bifidobacterium*, compared to the HoPS and HePS types in *Lactobacillus* strain (Ruas-Madiedo, 2014). Leivers et al. (2011) have reported the hypothetical biosynthesis pathway based on one kind of EPS-unit of HMW-EPS in strain *B. animalis* subsp. lactis IPLA-R1, which mainly included three steps: activated precursor’s synthesis and EPS-unit formation, export-polymerization process and chain length determination.

In this study, we isolated a new probiotic and EPS-producing *Bifidobaccterium*, named *B. breve* lw01. After genome sequencing and *in silico* analysis, a new EPS cluster and the hypothetical synthesis pathway were discovered. The extraction of EPS, purity determination and scanning electron microscopy observation confirmed its morphology, thus determining the composition of this EPS. Finally, we focused on its anti-tumor properties and preliminary investigated its mechanism. This study provided genetic information on EPS cluster from new isolated EPS-producing *Bifidobacterium*, connecting it with biosynthesis pathway and exploring its anti-tumor activities. Biochemical evidence was shown to explain its anti-tumor activity. Our research provided a theoretical basis for further application of this bifido-EPS in the probiotic field.

## Materials and Methods

### Isolation of Bacteria Stains

Fresh infant fecal sample was collected and incubated in a modified MRS-C liquid medium (MRS medium plus 0.5% cysteine • HCl) for 24 h. The medium was then transferred into a modified MRS-C agar plate and cultured at 37°C in anaerobic conditions for 48–72 h. Next, the different morphologies were picked up, and viscous colonies were separately inoculated for pure culture. Staining and morphological characteristics of the isolated bacteria were tested by microscopy. The protocol was approved by the Biomedical Ethics Committee of Peking University School and Hospital of Stomatology. Written informed consent was obtained from parents of the infant.

### Cell Lines and Culture Conditions

Human Head and Neck Squamous Cell Carcinoma cell line SCC15 (ATCC^®^ CRL-1623^TM^), CAL 27 (ATCC^®^ CRL-2095^TM^) and WSU-HN6 (obtained from Central Laboratory of Peking University School and Hospital of Stomatology) were used in this study. SCC15 were cultured in a 1:1 mixture of DMEM and Ham’s F12 medium (Life technology, Carlsbad, CA, United States) containing 10% FBS (Invitrogen, Waltham, MA, United States), 0.2% hydrocortisone (Selleck Chemicals, Houston, TX, United States) and 1% Penicillin/Streptomycin (Gibco, Waltham, MA, United States). CAL27 and WSU-HN6 were cultured in DMEM medium containing 10% FBS and 1% Penicillin/Streptomycin. All the cells were kept in a humidified atmosphere containing 95% air/5% CO_2_ at 37°C. For the anaerobic culture, we used the chamber with 93% N_2_/5% CO_2_/2% O_2_.

### Genomic DNA Isolation, Sequencing and Analysis of EPS Cluster in *Bifidobacterium*

Genome DNA was isolated using QIAamp DNA Mini Kit (Cat. 51304, QIAGEN, MD, United States) according the manufacturer’s instructions. 16s rRNA PCR was used to verify the purified strain type. The whole genome was sequenced on Illumina Hiseq 2000 platform (2 × 100 bp) second-generation sequencing platform. Sequence blast searches were performed on National Center for Biotechnology Information website^1^. The high-quality reads were assembled by SOAPdenovo. Based on the comparison-selected results, the relative abundance of different functional levels and EPS cluster searchers were investigated using KEGG (Kyoto Encyclopedia of genes and genomes), RAST (Rapid Annotation Subsystem Technology) and COG database. The genomes and EPS cluster information available in the GenBank database were used for the comparative analysis among *B. breve* lw01 and several *Bifidobacterium* strains.

### Extraction and Purification of EPS

For EPS extraction, purified bacteria were anaerobically cultured in 1 L of 10% skimmed milk at 37°C for 48 h. To isolate the crude EPS from the culture broth, 1 L of culture samples were centrifuged at 12,000 ×*g* for 15 min at 4°C. After obtaining the supernatant, TCA was added to a final concentration of 10% for 12 h at 4°C. Precipitated proteins were then removed by centrifugation at 4000 ×*g* for 20 min at 4°C. The EPS was precipitated from the supernatant with 3 volumes of cold ethanol followed by an overnight incubation at 4°C. After centrifugation at 6000 ×*g* for 30 min at 4°C, the pellet containing EPS was resuspended in 2 mL of distilled water and dialyzed (molecular weight cut-off: 6000–8000 Da) against 1 L of distilled water for 2 days with three water changes per 8 h.

Ion exchange chromatography of DEAE Sepharose Fast Flow (16 × 25 mm, GE Healthcare, Amersham, Uppsala, Sweden) was used for further EPS purification. Distilled water was applied to keep the pH of the ion exchange column neutral, eluted with 0.1, 0.2, 0.3, 0.4, and 0.5 M NaCl. The loading volume was 1 mL and the flow rate was 1 mL/min. Stepwise collection of 5 mL was applied for each tube. Then, the purified EPS from ion exchange chromatography was further purified by gel filtration chromatography of Sepharose CL-6B (GE Healthcare, Amersham, Uppsala, Sweden). Elution was performed with distilled water at a flow rate of 1 mL/min. The final eluted EPS was freeze-dried and stored at 4°C until analysis.

A HPLC (Agilent, Santa Clara, CA, United States) equipped with a NH_2_ column (Agilent, Santa Clara, CA, United States) was used for EPS further purification. The mobile phase consisted of 80% ammonium acetate solution and 20% acetonitrile at a flow rate of 1.0 mL/min, and the column temperature was kept on 30°C. The refractive index detector (RID) was used to detect the EPS.

The amount of EPS was determined using the phenol/sulfuric acid method. Glucose was used as the standard sugar. After the color reaction of the glucose standard solution with phenol/sulfuric acid, its absorbance was measured at 490 nm, and the OD_490_-standard sugar concentration curve was drawn. The diluted sample of EPS solution was examined using the same color reaction. The 490 nm absorbance value was measured, and the corresponding sugar concentration was found on the standard curve. The sugar concentration of the sample to be tested was calculated.

### Scanning Electron Microscopy

Scanning electron microscope was used to examine the morphology of the EPS. Briefly, the freeze-dried EPS powder was mounted on specimens slide, sputter coated with gold and examined using SEM (SEM; Hitachi, S-4700, Hitachi Ltd., Tokyo, Japan) at accelerating voltages of 10 and 15 kV, respectively.

### Monomer Composition of EPS

The monosaccharide standards dried to constant weight were weighed separately, and L-Rha, L-Ara, D-Gal, D-Glc, D-Man, and D-Fru prepared at different concentrations (0.2, 0.5, 1.0, 4.0, and 10.0 μg/mL) were mixed to standard solution. Standard curve for each monosaccharide was drew with monosaccharide concentration (μg/mL) as the abscissa (x) and peak area (nC × min) as the ordinate (y). 200 μL concentrated H_2_SO_4_ was added to 5 mg EPS and was left to stand still. Then 800 distilled water was added and hydrolyzed at 100°C for 2.5 h. After cooling, add to 2 mL and neutralize with solid BaCO_3_. Everything was centrifuged at 5000 r/min for 10 min to remove impurities, and remove the supernatant. The supernatant was diluted 20 times, filtered through a 0.20 μm microporous membrane and subjected to test.

High pH anion exchange chromatography with pulsed amperometric detection (HAPEC-PAD) was used to determine monosaccharide composition on a DIONEX-2500 ion chromatograph system, equipped with on-line automatic degassing GS50 quaternary gradient pump, ED50A electrochemical detector (pulse amperometry detection), Au working electrode and Ag/AgCl reference electrode. Chromatographic conditions were set as follows: sugar analysis column (CarboPac^TM^ PA10, 4 × 250 mm); sugar protection column (CarboPac PA10, 4 × 50 mm); mobile phase: A. H_2_O, B. 200 mmol/L NaOH, A:B = 91:9; injection volume: 25 μL; flow rate: 1.0 mL/min; column temperature: 30°C. Chromeleon 6.5 chromatography was used as workstation.

### Cell Proliferation and Cytotoxic Analysis

The EPS effect on cell proliferation and cytotoxicity were examined using CCK-8 assay and DAPI staining. Appropriate number of cells in 100 μL suspension were plated into 96-well plate and incubated in normoxic or hypoxic environment descripted above. After each time point, 10 μL of the CCK-8 solution (Bimake, Houston, TX, United States) was added to each well and incubated for another 4 h at 37°C. The absorbance at 450 nm was determined using a microplate reader. Cell nuclear morphology was stained by DAPI (Zhongshan Golden Bridge Biotechnology Co., Beijing, China). Each sample had three replicates and each experiment was run in triplicate.

### Western Blot

After treating cells with EPS, cells were harvested and lysed in RIPA buffer (Applygen Technology, Beijing, China) containing proteinase inhibitors and phosphatase inhibitors (ROCHE, Basel, Switzerland). BCA kit (Thermo Fisher Scientific, Rockford, IL, United States) was used to measure the protein concentration. Total of 70 μg amounts of protein samples were separated by 10% sodium dodecyl sulfate–polyacrylamide gel electrophoresis (SDS-PAGE) and transferred to polyvinylidene difluoride (PVDF) membranes by wet blotting. The membranes were blocked in 10% non-fat dry milk for 1 h and then probed with antibodies against MCM2 (1:1,000, ABclonal, Boston, MA, United States), Caspases-3 (1:1,000, CST, Boston, MA, United States), PARP (1:1,000, CST, Boston, MA, United States), cleaved-PARP (1:1,000, CST, Boston, MA, United States) and RPS18 (1:1,000, ABclonal, Boston, MA, United States) separately at 4°C overnight. Followed by incubation with peroxidase-linked secondary antibodies (1:10,000, CST, Boston, MA, United States) for 1 h at room temperature, the enhanced chemiluminescent (ECL) reagent (Thermo Fisher Scientific, Rockford, IL, United States) was used to visualize the immunoreactive proteins.

### Statistical Analysis

The absorbance value in CCK-8 assay and western blot results were expressed as means ± standard deviation (SD). One-way ANOVA was used for comparison among different concentration group and Fisher’s least significant difference (LSD) method was used for multiple comparisons in CCK-8 assay. Independent sample *t-*test was used to compare the treatment group and control group in western blot assay. All calculations and analyses were performed using SPSS 20.0 software (SPSS Inc., Chicago, IL, United States). A *P*-value < 0.05 was considered statistically significant.

## Results

### Isolation of *Bifidobacterium*

A total of 653 strains bacteria were isolated from the infant fecal samples. Among those, 23 viscous strains were isolated and transferred in a modified MRS-C medium. One strain was typically rod-shaped or bifurcated, non-spore-forming. Consequently, this clone was analyzed using 16S rRNA PCR (16S rRNA-F: 5′-AGAGTTTGATCMTGGCTCAG-3′ and 16S rRNA-R: 5′-TACGGYTACCTTGTTACGACTT-3′). The 16S rRNA sequence analyses showed 99% identity with *B. breve* DSM20213 (Supplementary Figure S1).

### Genome Information of *B. breve* lw01

**FIGURE 1:**
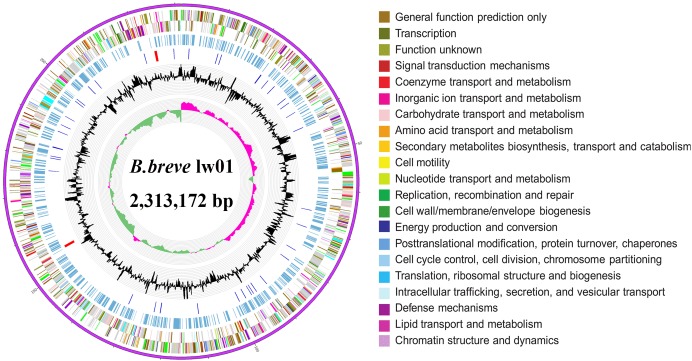
Circle genome map of *B. breve* lw01. Genome sequences (ring 1). COG Annotated coding sequences (ring 2 and 3), KEGG enzymes (ring 4), RNA genes (ring 5), GC content (ring 6), and GC skew (ring 7) are shown. Very short features were enlarged to enhance the visibility. Clustered genes, such as several rRNA genes, may appear as one-line due to space limitations. Image was created by the software Circos.

The complete genome sequence of our new isolated *B. breve* strain was submitted to GenBank under the accession Number CP034192. The complete genome of this strain is composed of one circular chromosome of 2,313,172 bp with a GC content of 58.7%, which is similar to other reported *B. breve* strains (GC content of 58.5–62.8%). In addition, the new strain contains a total of 63 RNAs including 54 tRNA genes, 2 rRNA, and 3 ncRNAs. A total of 1862 coding genes out of 2077 total genes were classified into 23 COG categories (Tables 1, 2 and Figure 1). The genome comparative analyses among *B. brev*e lw01 and other seven *Bifidobacterium* strains are presented in Supplementary Table S1. Consequently, this strain, named *B. breve* lw01 was deposited in China Center of Industrial Culture Collection (CICC) under the number CICC 24633.

**Table 1:** Features of *B. breve* lw01 genome.

Features	Chromosome
Genome size (bp)	2,313,172
GC content (%)	58.7
RNAs	63
rRNAs	2, 2, 2 (5S, 16S, 23S)
tRNAs	54
ncRNAs	3
CDSs (with protein)	1862


**Table 2:** COG categories of coding proteins in *B. breve* lw01 genome.

COG	Name	Count	Percent (%)
A	RNA processing and modification	2	0.10
C	Energy production and conversion	53	2.71
D	Cell cycle control, cell division, chromosome partitioning	31	1.59
E	Amino acid transport and metabolism	209	10.69
F	Nucleotide transport and metabolism	80	4.09
G	carbohydrate transport and metabolism	253	12.94
H	Coenzyme transport and metabolism	77	3.94
I	Lipid transport and metabolism	54	2.76
J	Translation, ribosomal structure and biogenesis	167	8.54
K	Transcription	153	7.83
L	Replication, recombination and repair	90	4.60
M	Cell wall/membrane/envelope biogenesis	106	5.42
N	Cell motility	12	0.61
O	Posttranslational modification, protein turnover, chaperones	74	3.79
P	Inorganic ion transport and metabolism	94	4.81
Q	Secondary metabolites biosynthesis, transport and catabolism	21	1.07
R	General function prediction only	153	7.83
S	Function unknown	93	4.76
T	Signal transduction mechanisms	92	4.71
U	Intracellular trafficking, secretion, and vesicular transport	15	0.77
V	Defense mechanisms	67	3.43
W	Extracellular structures	4	0.20
X	Mobilome: prophages, transposons	55	2.81


### *In silico* Analysis of Bifido-EPS Cluster

**FIGURE 2:**
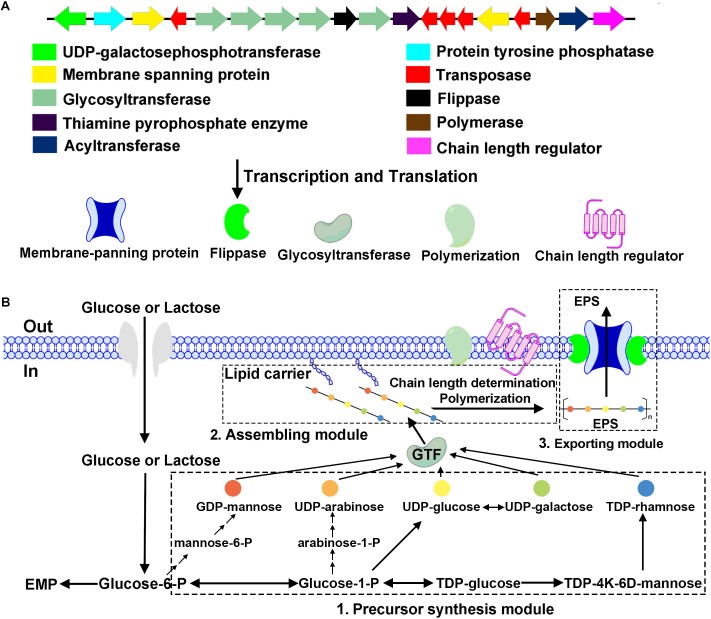
Physical map of the putative EPS cluster of *B. breve* lw01 and a schematic diagram of EPS biosynthesis in this strain. **(A)** The genes were categorized according to their potential functions, which are indicated with the colored arrows. **(B)** Hypothetical EPS biosynthesis pathway in strain *B. breve* lw01 based on its homology analysis of the EPS cluster.

*Bifidobacterium breve* lw01 harbors an EPS-encoding cluster with 14 predicted genes and 5 transposase coding genes. Priming-GTF (*p-gtf*) is the enzyme that catalyzes the initial step of EPS-unit synthesis; consequently, we first found this enzyme: UDP-galactosephosphotransferase (EH245_02110) coding for proteins of 576 amino acids. Five genes were coding for the GTFs (EH245_02130, EH245_02135, EH245_02140, EH245_02145, EH245_02155). Two types of genes could be responsible for transporting of the repeat EPS-units across the cytoplasmic membrane: flippase (EH245_02150) and membrane spanning protein (EH245_02120, EH245_02180). The chain length regulation and polymerization system were also found in this EPS-unit, which included the polymerase (EH245_02190), chain length regulator (EH245_02200) and the protein tyrosine phosphatase (EH245_02115). Another discovered feature was the presence of transposase (EH245_02125, EH245_02165, EH245_02170, EH245_02175, EH245_02185), an enzyme participated putative horizontal transfer of EPS among different taxa. There were also several genes coding for the precursor involved in the biosynthesis of EPS-unit: thiamine pyrophosphate enzyme (EH245_02160) and acyltransferase (EH245_02195) (Figure 2A).

**FIGURE 3:**
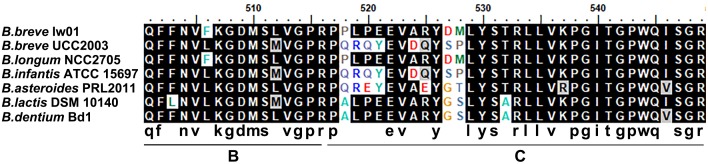
Alignment of an fragment of amino acid sequence of priming-GTF in *B. breve* lw01 and other *Bifidobacterium* strains. B at the bottom represented blocks involved in interaction with lipid carrier and C represented blocks involved in sugar-specific recognition.

We compared the EPS cluster information with other seven *Bifidobacteium* strains (Supplementary Table S1). Interestingly, the EPS cluster region has a G+C DNA content (49.7%) markedly lower than the *B. breve* strains (GC content of 58.5–62.8%), which inferred that it may be acquired by horizontal DNA transfer. The comparison of amino acid sequences of *p-gtf* showed that it was quite similar to the strain *B. longum* NCC2705 (with 98% identity). Partial amino acid identity of *p-gtf* among *B. breve* lw01 and other seven *Bifidobacterium* stains is shown in Figure 3. The results showed that they process the similar sequences particularly in the carboxy terminus regions.

### Hypothetical Pathway of EPS Biosynthesis in *B. breve* lw01

The hypothetical pathway of EPS biosynthesis in *B. breve* lw01 was divided into three modules. First module was the precursor synthesis. After glycose and lactose fluxed into the cytoplasm, activated NDP-sugar precursors modules were formed from the intermediate molecules of glycolysis. Second module was the assembling module. EPS-unit was catalyzed by *p-gtf* enzyme (UDP-galactosephosphotransferase). With the help of *p-gtf* (UDP-galactosephosphotransferase), one sugar-1-phosphate was transferred to the lipophilic carrier molecule and anchored to the cell membrane. Other five glycosyltransferases (GTFs) catalyzed the formation of glycosidic bonds by transferring new sugar moieties from a donor nucleotide sugar to the initial monosaccharide of the unit. A chain length regulator and polymerization system then assembled the EPS-lipophilic carrier unit and formed the finished EPS-unit. In the third exporting module, flippase and membrane spanning protein helped this finished EPS-unit to cross the cytoplasmic membrane to the extracellular face and build the final EPS polymers (Figure 2B).

### Extraction, Purification and Analysis of EPS

**FIGURE 4:**
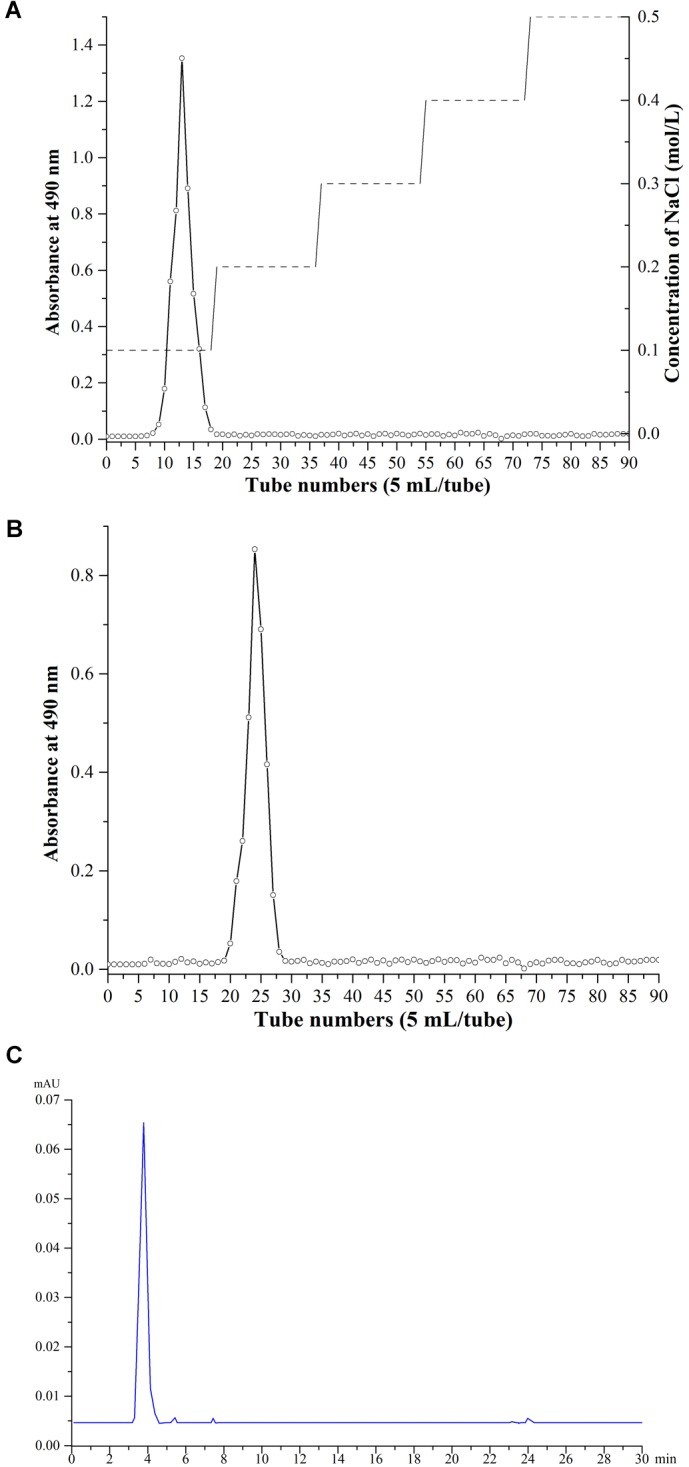
Purification and analysis of EPS. **(A)** Ion exchange chromatography. **(B)** gel filtration chromatography purification. **(C)** HPLC purification.

Crude EPS extracted from 1 L of 10% skimmed milk was about 80 mg. Only one kind of EPS fraction was obtained from Ion exchange chromatography with 0.1 mol/L NaCl solution, which appeared at 8–18 tubes (about 50 mL). When the elution tubes exceeded 50 tubes and the concentration of NaCl was above 0.3 M, no obvious polysaccharide component was eluted. After Ion exchange chromatography, we yielded about 26 mg EPS. After gel filtration chromatography and HPLC purification, 5 and 1 mg EPS were obtained, respectively. The single and symmetric peak showed that the EPS was purified with high quality (Figure 4).

### Scanning Electron Microscopy Images of EPS From *B. breve* lw01

**FIGURE 5:**
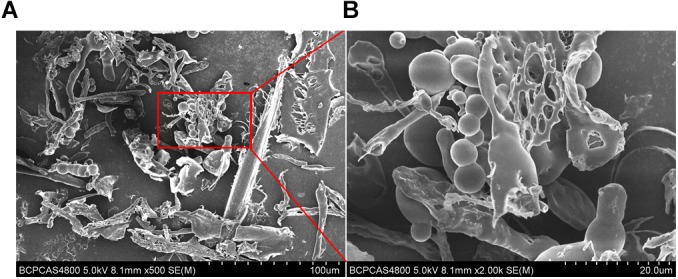
Scanning electron microscope images of purified EPS from *B. breve* lw01. **(A)** Magnification 500×. **(B)** In part magnified of A (magnification 2000×).

Scanning electron microscope is a useful method to investigate the surface and three-dimensional morphology of biomacromeolecules. The morphology of EPS observed by SEM showed that EPS was mainly composed of freely distributed irregular spherical bodies, flakes and nets. EPS was relatively rough, and was composed of depressions and voids, which may be due to the nature and structure of EPS, and the formation of branches. The use of different methods in the process of extraction and purification of EPS could cause different ultrastructure (Figure 5).

### Chemical Composition of *B. breve* lw01’s EPS

**FIGURE 6:**
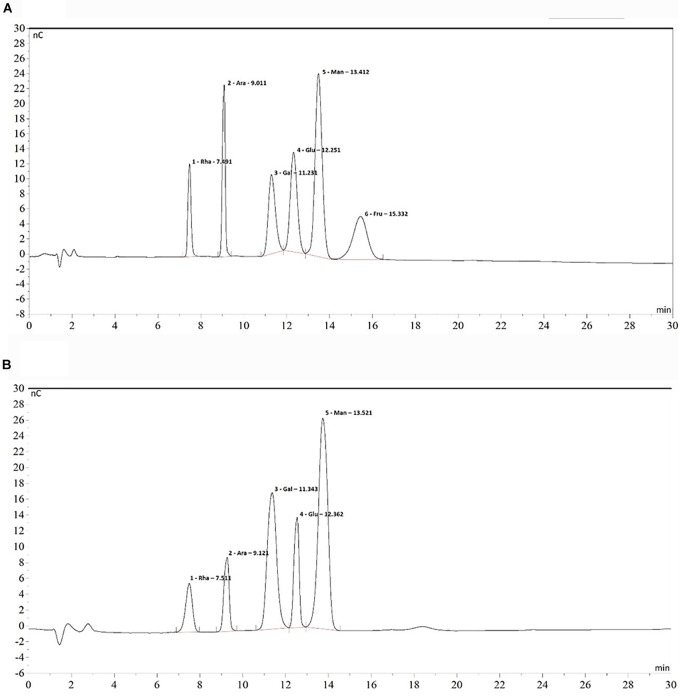
Chromatogram of standard monosaccharide **(A)** and EPS monosaccharide compositions **(B)** on HPAEC-PAD. From left to right: 1. Rhamnose 2. Arabinose 3. Galactose 4. Glucose 5. Mannose 6. Fructose.

The monomer composition of *B. breve* lw01’s EPS was determined by HPAEC-PAD. The results of monomer analysis indicated that EPS was a heteropolysaccharide mainly composed of rhamnose, arabinose, galactose, glucose and mannose in a molar ratio of 0.35:0.44:1.38:0.67:1.65 compared with the HPAEC spectra of standard monosaccharides (Figure 6). The constitution of our EPS was similar to the EPS composition produced by *B. animalis* RH (Shang et al., 2013).

### Anticancer Property of *B. breve* lw01’s EPS on HNSCC Cell Lines

#### Anti-proliferation Effect in Aerobic and Anaerobic Environment

**FIGURE 7:**
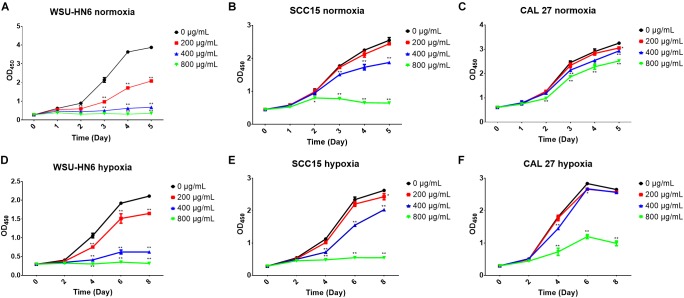
Cell proliferation inhibition of EPS from *B. breve* lw01 by CCK-8 assay. **(A–C)** Three HNSCC cell lines were cultured in normoxia condition and treated by gradient concentration of EPS from *B. breve* lw01. **(D–F)** Three HNSCC cell lines were cultured in hypoxia condition. All the experiments were run in triplicate. ^∗^*p* < 0.05; ^∗^*p* < 0.01.

**FIGURE 8:**
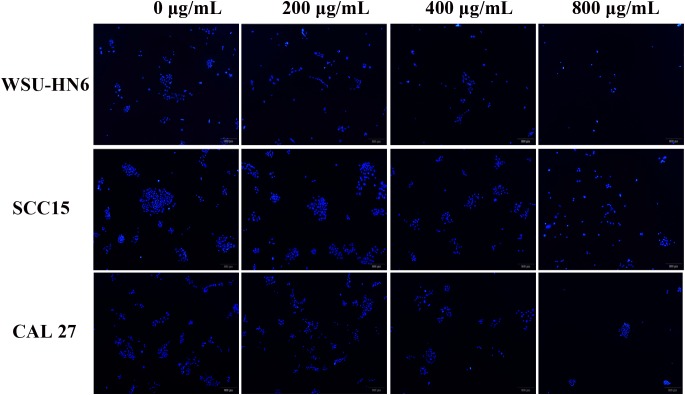
DAPI staining images of cell nuclear after treated with gradient concentration of EPS. All the cells were cultured in normoxia condition. magnification 4×.

CCK-8 assay and DAPI staining were used to examine the anti-tumor effect of EPS on HNSCC cell lines. CCK-8 results showed that EPS could inhibit cell proliferation in a concentration dependent manner. However, different cell line showed different sensitivity to EPS. The highest effect was observed in WSU-HN6, followed by SCC15 and CAL 27. On day 5, EPS inhibited the growth of WSU-HN6 by 46% at 200 μg/mL in aerobic environment. For CAL 27 cell line, in normoxia condition, 800 μg/mL EPS could inhibit CAL 27 cell line proliferation slightly, while the proliferation rate drastically decreased when culturing cells under hypoxic condition. This strengthened effect could be observed in the other cell lines as well (Figure 7). Furthermore, DAPI staining of cell nuclear with different treatment concentration presented the same tendency when cell lines were cultured in normoxia condition (Figure 8).

#### Cell Cycle and Apoptosis-Associated Protein Expression

**FIGURE 9:**
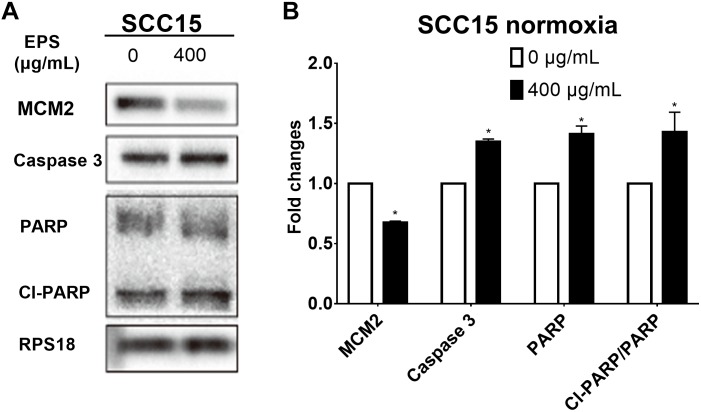
Cell cycle and apoptosis protein expression after treating SCC15 cell line with 400 μg/mL EPS. **(A)** Western blot assay. **(B)** Relative fold changes by calculating relative gray value using software Quantity One. ^∗^*p* < 0.05.

In order to find the detailed mechanism on the anti-proliferation effect of EPS, we tested the expression of cell-cycle and apoptosis associated protein by western bolt assay in a represented cell line SCC15 in normoxia condition. Decreased expression of MCM2 suggested that the cell cycle arrest occurs at G1-S phase. Increased expression of cell apoptosis protein caspase 3, PARP and the proportion of Cl-PARP/PARP inferred that EPS could promote the cell apoptosis (Figure 9).

## Discussion

Currently, EPS obtained from probiotic bacteria *Bifidobacterium* have been gaining increasing attention, mainly due to their role in immune system and gut microbiota modulation. In the present study, we isolated a new *B. breve* strain from infant feces. After sequencing its genome, we predicted its EPS cluster and hypothetic synthesis pathway. Based on the EPS extraction and high-quality purification, we determined its chemical composition. Finally, we focused our attention on its anticancer activity and provided biochemical evidence to explain it. Thus, this study provided a better understanding of the function-genome cluster relation and biosynthesis in bifido-EPS, which could facilitate genetic and metabolic engineering indispensable for production of tailor-made EPS used in food or drug industry.

Extraction and purification were necessary to examine the biological activity of *B. breve* lw01’s EPS. The yield of crude EPS released to culture medium can be influenced by environmental and nutritional conditions, such as the medium composition, pH, temperature, oxygen and the growth phase (Leroy and De Vuyst, 2016; Bengoa et al., 2018). In our study, slimmed milk was chosen for the initial cultivation to avoid sugar in the medium that interferes with the EPS extraction. Crude bifido-EPS was followed by the further purification (Ion exchange chromatography and gel filtration chromatography), which yielded high purity bifido-EPS, thus providing good research basis for the biological activity. Multiple purification induced the poor yield, which was affected by many factors such as EPS sample loading volume, eluent and elution rate. In the course of the research, we observed that the low purification efficiency affected our further research. Therefore, future studies should focus on the elevation of the purification efficiency of EPS isolation.

Previous studies have indicated that genes responsible for the biosynthesis of EPS are presented in the form of clusters in *Bifidobacterium* genome (Leivers et al., 2011; Fanning et al., 2012; Hidalgo-Cantabrana et al., 2014). Due to the high interspecies variability existence, and deep excavation of genome sequences from different database, the comparison-selected results were used to examine the gene cluster responsible for EPS synthesis. We find complete EPS gene cluster was involved in the biosynthesis of EPS and the gene cluster could be divided into three following groups: the gene responsible for repeated unit synthesis, polymerization/chain length determination and exportation. No transcriptional regulator was observed as in other *Bifidobacterium* (Ruas-Madiedo, 2014). The EPS biosynthesis pathway was similar but not identical to that reported in *B. breve* UCC2003. In *B. breve* UCC2003, the EPS synthesis was bidirectional and responsible for production of one or two polymers (Fanning et al., 2012). For the further confirmation of the exact function of each gene involved in our bifdo-EPS gene cluster, mutants by deletion part of the EPS clusters could be used by comparing to their counterparts.

Among the biosynthesis pathway, repeated unit synthesis was the key step to determine the final EPS-unit constitution and GTF was the key enzyme involved in this part. The amino acid identity of *p-gtf* among *B. breve* lw01 and other seven *Bifidobacterium* stains was lower. The similar sequences were observed, particularly in the carboxy terminus regions, due to the presence of regions involved in interaction with lipid carrier (region B) and sugar specificity (region C) (Provencher et al., 2003; Yan et al., 2017). However, the composition and the structure of bifido-EPS have not yet been fully illustrated for any of the strains containing completed genomes (Hidalgo-Cantabrana et al., 2014). Therefore, although the numbers and specificities of GTFs could contribute to the different EPS-unit constitution and structures, it is still not possible to fully establish the association between the genetics and physico-chemical characteristics (Hidalgo-Cantabrana et al., 2014). Moreover, in this work, we did not focus on the structure of EPS. Accordingly, future studies should focus on investigating the genetics and physico-chemical correlation based on EPS genome-composition-structure characters.

In our previous study, we already verified that the strain *B. breve* possess an anti-tumor effect on HNSCC tumor-bearing model (Wang et al., 2017). Yet, the exact mechanism was not fully explored. Existing studies have shown that the presence of bacterial molecules structure (DNA, proteins, EPS or metabolites) could have a specific effect on the immune or inflammation system (Menard et al., 2010; Hevia et al., 2014; Hidalgo-Cantabrana et al., 2014). In the present study, we further examined whether the new extracted bifido-EPS may be responsible for the anticancer bioactivity.

Currently, there are only few studies on the anticancer effect of EPS from *Bifidobacterium* strains. *In vitro* experiments have shown that polysaccharide fraction extracted from *B. bifidum* BGN4 could inhibit the growth of colon cancer cell lines HT-29 and HCT-116, but had no effect on Caco-2 cell line. Previous study has reported that the inhibition rate on HT-29 cell line was 50.5 ± 3.6% at 80 μg/mL by Trypan blue exclusion assay. It is speculated that the anticancer effect may be related to the inhibition of DNA synthesis and reduction of cell proliferation capacity (You et al., 2004). The research from Nam Joo Ha’s group showed that butanol extract of *B. adolescentis* SPM0212 could inhibit the growth of Caco-2, HT-29, and SW480 cells by 70, 30, and 40%, respectively, at 200 μg/mL. The mechanism may rely on inducing macrophage activation and increasing the production TNF-α and NO (Lee et al., 2008). In the present study, we investigated anticancer effect of bifido-EPS on HNSCC cell line, which exerted proliferation inhibition effect in dose-dependent manner. In addition, different cells presented different sensitivity to bifido-EPS.

As for the mechanisms of the anti-cancer property of EPS, except for the inhibition of DNA synthesis and immune modulation reported above, EPS has anti-oxidative properties and can inhibit the expression of gene involved in angiogenesis (Deepak et al., 2016). Cell proliferative activity is closely related to the cell cycle, which is regulated by the DNA replication initiation factor. MCM 2, known as DNA replication licensing factor, is involved in pre-replicative complex formation during G1 phase and it allows for the DNA replication initiation in the subsequent S-phase (Gambus et al., 2011). Downregulation of MCM2 could increase cell death when the cells encounter replication stress during S-phase (Ibarra et al., 2008). MCM2 has also been applied as a proliferation marker in many types of cancer (Tan et al., 2001; Yousef et al., 2017; Zhou et al., 2018). In addition, its downregulation is considered as an attractive target in tumor chemotherapy (Simon and Schwacha, 2014). In this study, the expression of MCM2 was downregulated, which suggested that EPS could arrest cell cycle in G1-S phase in HNSCC cell line. PARP is activated when cells sustained DNA damage and it is the main target of caspase 3, whose activation could mediate a latent specific proteolytic cleavage in apoptosis (Brauns et al., 2005; Yu et al., 2006). In our study, EPS increased the expression of caspase 3 and the cleaved-PARP proportion, while inferred cell apoptosis occurred in treatment group.

The chemical composition of our EPS, which included rhamnose, arabinose, galactose, glucose, and mannose, could explain its anti-tumor effect to some extent. It is quite interesting that mannose accounted for the largest proportion of the bifido-EPS, which have been reported to have a significant anti-tumor activity. Mannose could impair tumor growth by inhibiting the proliferation while promoting the apoptosis of cancer cell. It also revealed chemo-sensitization character. Administration of doxorubicin with mannose showed an obviously enhanced anti-tumor effect (Gonzalez et al., 2018). Moreover, the level of mannose in serum is associated with the prognosis of esophageal adenocarcinoma patients. The patients with low level of d-mannose have higher risk of recurrence and death (Gu et al., 2017). Besides, rhamnose has also been reported to have anti-tumor activity, since it can enhance the immunogenicity of melanoma-associated antigen A3, which stimulates antitumor immune responses (Zhang et al., 2016). The galactose is widely used backbone of the nanocarrier in cancer therapy, beneficial for targeted therapy of tumors (Jain et al., 2018). When conjugated with platinum (II), the complex has well therapeutic and target effect (Wu et al., 2016). In our next study, we plan to further investigate the effect of our bifido-EPS *in vivo*.

To sum up, the current work isolated one new probiotic strain *B. breve* lw01 and sequenced its genome, which led to discovery of a new EPS biosynthesis cluster and prediction of its biosynthesis pathway. After extraction and purification, high purity of our EPS showed to possess an anticancer activity in HNSCC cell lines. In addition, the composition of bifido-EPS showed that mannose accounted for the largest proportion. The preliminary mechanisms could rely on its cell cycle arrest and promote cell apoptosis effect. We believe that this bifido-EPS could be used to facilitate genetic and metabolic engineering and to produce tailor-made EPS for further application in functional food or drug industry.

## Ethics Statement

The protocol was approved by the Biomedical Ethics Committee of Peking University School and Hospital of Stomatology. Written informed consent was obtained from parents of the infant.

## Author Contributions

GL and CG contributed the conception and design of the study. LW and KT organized the database and analysis EPS cluster information. YW, QL, and LX carried out the study. YW performed the statistical analysis and wrote section of the manuscript. LW wrote the first draft of the manuscript. All authors contributed to manuscript revision, read and approved the submitted version.

## Conflict of Interest Statement

The authors declare that the research was conducted in the absence of any commercial or financial relationships that could be construed as a potential conflict of interest.

## Footnotes

1 http://www.ncbi.nlm.nih.gov

## Abbreviations

bifido-EPS: *Bifidobacterium*-EPS
COG: clusters of orthologous groups
EPS: exopolysaccharide
HAPEC-PAD: High pH anion exchange chromatography with pulsed amperometric detection
HePS: heteropolysaccharides
HNSCC: head and neck squamous cell carcinoma
HoPS: homopolysaccharides
HPLC: high-performance liquid chromatography
MCM2: mini-chromosome maintenance 2
*p-gtf*: priming-GTF
SEM: scanning electron microscope
TCA: trichloroacetic acid
