# Supplementary material for: Exopolysaccharide, Isolated From a Novel Strain Bifidobacterium breve lw01 Possess an Anticancer Effect on Head and Neck Cancer – Genetic and Biochemical Evidences
Source: Front Microbiol. 2019 May 9;10:1044. doi: 10.3389/fmicb.2019.01044 (PMC6520658; doi:10.3389/fmicb.2019.01044)
Supplement: Supplementary file 1 [file Table_1.docx]

***Supplementary Material***

**Table S1. Comparative analysis of genome and *eps* cluster characters among *B.breve* lw01 and several *Bifidobacterium* strains available in the GenBank database.**

|  |  |  |  |  |  |  |  |  |  |  |  |  |  |  |  |
| --- | --- | --- | --- | --- | --- | --- | --- | --- | --- | --- | --- | --- | --- | --- | --- |
| Species | Strains | Genome Accession No. | Genome | | | |  | eps cluster | | | | | | | Reference |
|  |  |  | Length (bp) | CDSs  (with protein) | G+C content (%) | rRNA |  | Length (bp) | Coding genes | G+C content (%) | No.  ME^a^ | Priming-GTF | | amino acid identity of rfbP % (to B.breve lw01) |  |
|  |  |  |  |  |  |  |  |  |  |  |  | *cpsD* | *rfbP* |  |  |
| *B.breve* | lw01 | CP034192 | 2,313,172 | 1,862 | 58.7 | 2, 2, 2 (5S, 16S, 23S) |  | 22,978 | 14 | 49.7 | 5 | ND | EH245_02110 | 100 | This research |
| *B.breve* | UCC2003 | CP000303 | 2,422,684 | 1,854 | 58.7 | 2, 2, 2 (5S, 16S, 23S) |  | 29,272 | 21 | 50.3 | 4 | ND | Bbr_0430 | 74 | (Fanning et al., 2012) |
| *B.longum* | NCC2705 | AE014295 | 2,256,640 | 1,729 | 60 | 4, 4, 4 (5S, 16S, 23S) |  | 34,380 | 15 | 55.4 | 6 | BL0237 | BL0249 | 98 | (Schell et al., 2002) |
| *B.infantis* | ATCC15697 | CP001095 | 2,832,748 | 2,423 | 59.86 | 4, 4, 4 (5S, 16S, 23S) |  | 19,311 | 13 | 51.4 | 5 | ND | Blon_2114 | 79 | (Sela et al., 2008) (Hidalgo-Cantabrana et al., 2014) |
| *B.asteroides* | PRL2011 | CP003325 | 2,167,304 | 1,658 | 60.49 | 2, 2, 2 (5S, 16S, 23S |  | 13,454 | 12 | 49.1 | 0 | ND | BAST_1667 | 45 | (Bottacini et al., 2012) |
| *B.thermophilum* | RBL67 | CP004346 | 2,291,643 | 1,845 | 60.1 | 4, 4, 4 (5S, 16S, 23S) |  | 27,116 | 20 | 52.9 | 3 | ND | D805_0348 | 54 | (Hidalgo-Cantabrana et al., 2014) |
| *B.lactis* | DSM10140 | CP001606 | 1,938,483 | 1,566 | 60.5 | 4, 4, 4 (5S, 16S, 23S) |  | 54,250 | 42 | 53.5 | 1 | Balat_1392 | Balat_1371 | 47 | (Barrangou et al., 2009; Hidalgo-Cantabrana et al., 2014) |
| *B.dentium* | Bd1 | CP001750 | 2,636,367 | 2,129 | 58.54 | 5, 5, 5 (5S, 16S, 23S) |  | 58,994 | 49 | 52 | 0 | ND | BDP_1857 | 52 | (Ventura et al., 2009)(Hidalgo-Cantabrana et al., 2014) |
|  |  |  |  |  |  |  |  |  |  |  |  |  |  |  |  |

a ME, Mobile Elements (transposase)

Barrangou, R., Briczinski, E.P., Traeger, L.L., Loquasto, J.R., Richards, M., Horvath, P., et al. (2009). Comparison of the complete genome sequences of Bifidobacterium animalis subsp. lactis DSM 10140 and Bl-04. *J Bacteriol* 191(13)**,** 4144-4151. doi: 10.1128/JB.00155-09.

Bottacini, F., Milani, C., Turroni, F., Sanchez, B., Foroni, E., Duranti, S., et al. (2012). Bifidobacterium asteroides PRL2011 genome analysis reveals clues for colonization of the insect gut. *PLoS One* 7(9)**,** e44229. doi: 10.1371/journal.pone.0044229.

Fanning, S., Hall, L.J., Cronin, M., Zomer, A., MacSharry, J., Goulding, D., et al. (2012). Bifidobacterial surface-exopolysaccharide facilitates commensal-host interaction through immune modulation and pathogen protection. *Proc Natl Acad Sci U S A* 109(6)**,** 2108-2113. doi: 10.1073/pnas.1115621109.

Hidalgo-Cantabrana, C., Sanchez, B., Milani, C., Ventura, M., Margolles, A., and Ruas-Madiedo, P. (2014). Genomic overview and biological functions of exopolysaccharide biosynthesis in Bifidobacterium spp. *Appl Environ Microbiol* 80(1)**,** 9-18. doi: 10.1128/AEM.02977-13.

Schell, M.A., Karmirantzou, M., Snel, B., Vilanova, D., Berger, B., Pessi, G., et al. (2002). The genome sequence of Bifidobacterium longum reflects its adaptation to the human gastrointestinal tract. *Proc Natl Acad Sci U S A* 99(22)**,** 14422-14427. doi: 10.1073/pnas.212527599.

Sela, D.A., Chapman, J., Adeuya, A., Kim, J.H., Chen, F., Whitehead, T.R., et al. (2008). The genome sequence of Bifidobacterium longum subsp. infantis reveals adaptations for milk utilization within the infant microbiome. *Proc Natl Acad Sci U S A* 105(48)**,** 18964-18969. doi: 10.1073/pnas.0809584105.

Ventura, M., Turroni, F., Zomer, A., Foroni, E., Giubellini, V., Bottacini, F., et al. (2009). The Bifidobacterium dentium Bd1 genome sequence reflects its genetic adaptation to the human oral cavity. *PLoS Genet* 5(12)**,** e1000785. doi: 10.1371/journal.pgen.1000785.
