# Supplementary material for: Exopolysaccharide, Isolated From a Novel Strain Bifidobacterium breve lw01 Possess an Anticancer Effect on Head and Neck Cancer – Genetic and Biochemical Evidences
Source: Front Microbiol. 2019 May 9;10:1044. doi: 10.3389/fmicb.2019.01044 (PMC6520658; doi:10.3389/fmicb.2019.01044)
Supplement: Supplementary file 2 [file Data_Sheet_1.PDF]

# *Supplementary Material*

CTCCGTAGAAAGGAGGTGATCCAGCCGCACCTTCCGGTACGGCTACCTTGTTACGACTTAGTCC
CAATCACGAGCCTCACCTTAGACGGCTCCCTCCCGCAAGGGGTTAGGCCACCGGCTTCGGGTG
CTGCCCACTTTCATGACTTGACGGGCGGTGTGTACAAGGCCCGGGAACGCATTCACCGCGACG
TTGCTGATTCGCGATTACTAGCGACTCCGCCTTCACGCAGTCGAGTTGCAGACTGCGATCCGAA
CTGAGACCGGTTTTCAGGGATCCGCTCCAGCTCGCGCTGTCGCATCCCGTTGTACCGGCCATTG
TAGCATGCGTGAAGCCCTGGACGTAAGGGGCATGATGATCTGACGTCATCCCCACCTTCCTCCG
AGTTAACCCCGGCGGTCCCCCGTGAGTTCCCGGCACAATCCGCTGGCAACACGGGGCGAGGGT
TGCGCTCGTTGCGGGACTTAACCCAACATCTCACGACACGAGCTGACGACGACCATGCACCAC
CTGTGAACCCGCCCCGAAGGGAAACCCCATCTCTGGGGTCGTCGGGAACATGTCAAGCCCAGG
TAAGGTTCTTCGCGTTGCATCGAATTAATCCGCATGCTCCGCCGCTTGTGCGGGCCCCCGTCAAT
TTCTTTGAGTTTTAGCCTTGCGGCCGTACTCCCCAGGCGGGATGCTTAACGCGTTAGCTCCGACA
CGGAACCCGTGGAACGGGCCCCACATCCAGCATCCACCGTTTACGGCGTGGACTACCAGGGTA
TCTAATCCTGTTCGCTCCCCACGCTTTCGCTCCTCAGCGTCAGTAACGGCCCAGAGACCTGCCTT
CGCCATTGGTGTTCTTCCCGATATCTACACATTCCACCGTTACACCGGGAATTCCAGTCTCCCCT
ACCGCACTCAAGCCCGCCCGTACCCGGCGCGGATCCACCGTTAAGCGATGGACTTTCACACCG
GACGCGACGAACCGCCTACGAGCCCTTTACGCCCAATAATTCCGGATAACGCTTGCACCCTAC
GTATTACCGCGGCTGCTGGCACGTAGTTAGCCGGTGCTTATTCGAAAGGTACACTCAACACAAA
ATGCCTTGCTCCCTAACAAAAGAGGTTTACAACCCGAAGGCCTCCATCCCTCACGCGGCGTCGC
TGCATCAGGCTTGCGCCCATTGTGCAATATTCCCCACTGCTGCCTCCCGTAGGAGTCTGGGCCG
TATCTCAGTCCCAATGTGGCCGGTCGCCCTCTCAGGCCGGCTACCCGTCGAAGCCATGGTGGG
CCGTTACCCCGCCATCAAGCTGATAGGACGCGACCCCATCCCATGCCGCAAAGGCTTTCCCAA
CACACCATGCGGTGTGATGGAGCATCCGGCATTACCACCCGTTTCCAGGAGCTATTCCGGTGCA
TGGGGCAGGTCGGTCACGCATTACTCACCCGTTCGCCACTCTCACCACCAGGCAAAGCCCGAT
GGATCCCGTTCGACTTGCATGTGTTAAGCACGCCGCCAGCGTTCATCCTGAGCCAGAATCGAAC
CCTCCACAAAA

**Figure S1.** The 16s rRNA sequence of *B.breve* lw01, which was 99% identity with *B. breve* DSM20213.
